# Serum amyloid protein A in inflammatory bowel disease: from bench to bedside

**DOI:** 10.1038/s41420-023-01455-5

**Published:** 2023-05-10

**Authors:** Rirong Chen, Qia Chen, Jieqi Zheng, Zhirong Zeng, Minhu Chen, Li Li, Shenghong Zhang

**Affiliations:** 1 Department of Gastroenterology, The First Affiliated Hospital, Sun Yat-sen University, Guangzhou, China; 2 Zhongshan School of Medicine, Sun Yat-sen University, Guangzhou, China

**Keywords:** Predictive markers, Immunological disorders

## Abstract

Inflammatory bowel diseases (IBD) is featured by gastrointestinal inflammation and a disease course with alternating recurrence and remission. The global burden caused by IBD has significantly boosted in recent years, necessitating treatment optimization. Serum amyloid A (SAA) is a class of 104 amino acid conservative acute-phase proteins, which is essential in immune-mediated inflammatory processes, like IBD. The SAA monomeric structure is composed of four α-helical regions and a C-terminal amorphous tail. Its disordered structure enables multiple bindings to different ligands and permits multiple functions. It has been proven that SAA has dual roles in the inflammatory process. SAA stimulates the pro-inflammatory cytokine expression and promotes the pathogenic differentiation of TH17 cells. In addition, SAA can remove toxic lipids produced during inflammatory responses and membrane debris from dead cells, redirect HDL, and recycle cholesterol for tissue repair. In IBD, SAA acts on gut epithelium barriers, induces T-cell differentiation, and promotes phagocytosis of Gram-negative bacteria. Owing to the tight connection between SAA and IBD, several clinical studies have taken SAA for a biomarker for diagnosis, assessing disease activity, and predicting prognosis in IBD. Furthermore, 5-MER peptide, a drug specifically targeting SAA, has shown anti-inflammatory effects in some SAA-dependent animal models, providing novel insights into the therapeutic targets of IBD.

## Facts

- Serum amyloid A (SAA) is a famous acute-phase protein and essential in the immune-mediated inflammatory process.
- SAA is demonstrated to be a promising biomarker in various immune-related diseases.
- Accumulative evidence show that SAA may participate in the pathogenesis of inflammatory bowel disease.

## Open questions

- What are the functions of SAA in the development and progression of inflammatory bowel disease?
- Can SAA be an effective biomarker for diagnosis, disease activity assessment and prognosis prediction in inflammatory bowel disease?
- Can SAA become a potential treatment target in inflammatory bowel disease?

## Introduction

Inflammatory bowel diseases (IBD), including Crohn’s disease (CD) and ulcerative colitis (UC), are chronic immune-mediated inflammatory diseases. IBD is characterized by relapsing and remitting disease courses and causes an undesirable living quality in patients [1]. Meanwhile, the incidence and prevalence of IBD have increased dramatically over the last 20 years, especially in newly industrialized regions [2]. For instance, the IBD incidence in Brazil sharply increased eight folds from 1986 to 2005, to 8.0 per 100,000 [3]. In Taiwan, the IBD prevalence also increased more than six folds from 2001 to 2015, to 12.8 and 3.9 per 100,000 for UC and CD, respectively [4]. Furthermore, it is foreseeable that in the coming period, the number of patients with IBD worldwide will continue to rise. This places a huge burden on both patients and the healthcare systems worldwide [5]. Unfortunately, no current drugs or treatment approaches can cure IBD, as the mechanisms of IBD development are still unclear [6, 7]. Therefore, it is important to completely understand the etiopathogenesis of IBD and identify effective therapeutic targets.

Serum amyloid A (SAA) is a highly conserved family of acute-phase response proteins, the pathophysiology of which has been studied for more than 60 years [8–10]. SAAs are mainly synthesized in the liver, but also widely expressed in other parts of the body, such as the stomach and the intestine [11]. In healthy individuals, the plasma level of SAA is very low. However, in emergency conditions, such as inflammation, trauma, and viral infection, the level of SAA in the body can increase rapidly, even up to 1000-fold [11–13]. Accordingly, it is believed that SAA has a variety of functions during the acute phase. In recent years, SAA has been demonstrated to participate in immune regulation, especially T-cell immunity [14, 15]. SAA can regulate innate and adaptive immunity as well as mediate lipid transportation during inflammation [13, 16–18]. Research has indicated the importance of SAA in the generation and progression of hepatitis C virus infection [19], cancers [20, 21], and IBD. In addition, SAA has been identified as a biomarker for IBD diagnosis, disease activity monitoring, and prognosis prediction. However, the role of SAA in IBD requires further elucidation.

We will briefly state the structure and function of SAA and explore its role in IBD pathogenesis in this review. In addition, we summarized the current researches on the applications of SAA in IBD patient management.

## SAA

### Structure of SAA

The human SAA family contains four conservative genes: SAA1-4, all located on chromosome 11p. SAA1 and SAA2 encode virtually identical homonymic 104 amino acid proteins [22]. SAA3 is thought to be a pseudogene [23], although one study suggests otherwise [24]. SAA4 is constitutively expressed, and owing to an eight amino acid sequence insertion, it is lengthened to 112 amino acids [25]. In mice, five SAA genes have been reported on chromosome 7, one of which is also a pseudogene, others correspond to SAA1/2. The SAA1-4 genes in mice are almost identical to those in humans, while the mice SAA3 gene can be properly transcribed and translated. Typically, SAA1-3 can all be detected in mice serum by anti-SAA antibodies, while in human serum only SAA1/2 are detected [13]. The amino acid sequences of SAA genes have been highly conserved during the evolution of mammals and other vertebrates. Although variations have been identified, they remain limited [13, 22]. This conservation of amino acid sequences has led to a steady physical structure of SAAs; thus, they reliably perform their functions [22].

The SAA family has a three-dimensional shape characterized by a monomer structure and a fiber structure with specific amino acid sequences. The high-resolution structure of human SAA1 was determined using X-ray crystallography [26]. The human SAA1 monomeric structure is composed of four α-helical regions and a C-terminal amorphous tail with no β-folded region [26, 27]. The instability of the first 13 residues of SAA is believed to be related to the formation of amyloid fibers [26, 28, 29]. Regarding the structure of SAA fibers, the most prominent feature is the stacked anti-parallel β-folded sheets, which can be parallel in some situations. These stacked sheets are typically 20 nm in diameter and 10 Å apart. Extensive van der Waals interactions and hydrogen bonding stabilize such a structure [30]. Most fibrils consist of approximately 76 N-terminal residues, while both the shorter and longer forms have been reported. Furthermore, substantial recombination must occur before SAA fiber assembly since no β-folded region in the maternal monomer [26, 27].

### Functions of SAA

The function of SAA is closely related to its structure. As mentioned above, the human SAA consists of four helices (h1-h4) and an amorphous C-terminal tail [26]. It was found that different sites of SAA can bind to different ligands and perform distinct functions [31, 32]. Specifically, h1 binds cholesterol, enhancing the absorption of cholesterol into cells [33]. H3 binds and transports retinol during bacterial infection [26, 34, 35]. The structure made up of h1 and h3 can bind reversibly to high-density lipoprotein (HDL), enabling SAA to mediate lipid transportation and act as a hub to facilitate molecular interactions during inflammation [31, 32, 35]. Another acidic structure consisting of h2 and h4 is thought to interact with the basic surface sites of cell scavenger receptor SR-B1 and its homologous receptors, which distribute on the surface of cells, like macrophages. This combination mediates the internalization of SAA and then simulates lipid isolation, removal and circulation for tissue repair [36–38]. In addition, the C terminus of SAA can bind to glycosaminoglycans, including heparin and heparan sulfate, and then influence a variety of proteins, such as apolipoprotein A1 [39, 40]. Moreover, this binding inhibits the combination of SAA with HDL, promoting the formation of amyloid fibrils [26]. Among these, the HDL combination is vital. During inflammation, several SAAs bind at the HDL through the h1-h3 sites, meanwhile, more ligands can still attach to the other sites of SAA [36, 41]. Thus, such complexes can act as pivots to facilitate the dynamic interactions of bound ligands, accelerating signaling networks [32]. A hypothesis has been proposed to explain how such a small protein binds to many types of ligands. This indicates that SAA has a disordered structure in the absence of ligand binding, and promotes interaction with various ligands by folding after ligand binding [32, 42].

**Fig. 1:**
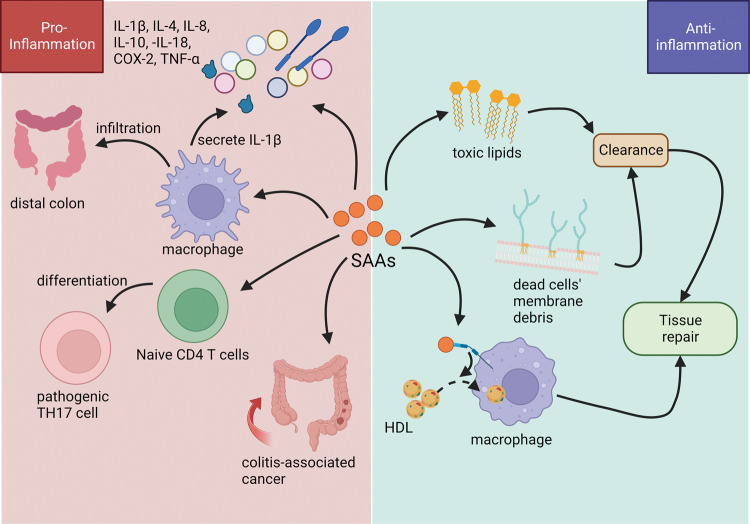
The multiple functions of SAA in inflammation. SAA could induce the secretion of pro-inflammatory cytokines, including IL-1β, IL-4, IL-8, IL-10, IL-18, COX-2, and TNF-α. SAA promotes the infiltration of macrophages in distal colon and the pathogenic differentiation of CD4 + T cells. Its binding to macrophages leads to IL-1β secretion and lipid internalization for tissue repair. Higher SAA level contributes to colitis-associated cancer. SAA also helps cleaning toxic lipids and membrane debris to accelerate tissue repair.

The expression level of SAA parallels the development of mucosal inflammation [43]. During the acute phase, the combination of SAA to toll-like receptors (TLRs) on innate immune cells facilitates inflammatory cytokines release, activates NF-κB, and promotes T-cell priming [31]. However, at the same time, by binding to TLR2 on myeloid-derived suppressor cells in mice spleen, SAA protects them from apoptosis induced by TNF-α, enabling them to promote macrophages transformation into anti-inflammatory phenotype and inhibit their pro-inflammatory transformation with the assistance of MyD88 and interferon regulatory factor 4, which helps inflammation resolving [31, 44]. Further investigations have also revealed the duality of SAA in the chronic inflammatory process (Fig. 1). In terms of inflammation promotion, studies have found SAA enhances the pro-inflammatory cytokines expression, which correlate to immune-related diseases, and can also promote macrophage activation and T lymphocyte differentiation. Specifically, SAA induces the expression of pro-inflammatory interleukin (IL)-8 and COX-2 [45]. SAA1/2 double-knockout mice showed higher stool consistency as well as lesser rectal bleeding and other histologic damage associated with colitis. SAA1/2 deficiency leads to descended concentrations of IL-10, IL-4 as well as tumor necrosis factor (TNF)-ɑ, and SAA3 is also down-regulated [21]. Studies have also found SAA promotes macrophage IL-1β release by stimulating caspase-1 activation mediated by the NLRP3 inflammasome, which can activate the MAPK and NF-κB signaling cascades, thereby promoting innate and adaptive immune responses [46–48]. Thereafter, excessive pores caused by inflammasome activation could promote pyroptosis, resulting in IL-18 and IL-1β secretion [49]. In addition, in SAA1/2 double-knockout mice with experimental autoimmune encephalomyelitis, their disease onsets were delayed. Although no delay is found in SAA3 knockout mice, they recovered faster, especially in the chronic stage. Inversely, more TH17 lymphocytes were found in the secondary lymphoid tissue of mice over-expressing SAA1, and these mice are more likely to develop such inflammatory diseases when compared with controls. Experiments suggested that SAA has vital pro-inflammatory effects [14]. Furthermore, a network analysis identified SAA1 expression as an essential indicator in IBD patients, associating with mucosal T lymphocytes, gut microbiota, and the tissue environment [15]. In this analysis, a direct positive correlation between SAA1 and T helper (TH) 17 cells was found [15]. Further research has indicated that SAA can induce the differentiation of naive CD4 T cells into pathogenic TH17 cells and promote their pathogenicity [14]. Moreover, SAA deficiency reduces colitis-associated cancers [21]. In terms of inflammation resolution, in vitro experiments have shown that SAA could automatically pack lipids into nanoparticles in the neutral environment [50–52]. Such a function, on the one hand, enables SAA to clean toxic lipids produced during inflammatory responses [53], compensating for the lack of albumin during acute inflammation [54]. Besides, it permits SAA-only lipoproteins to separate membrane debris from dead cells for clearance, minimizing tissue damage [36, 55–57]. Such function is significantly improved during the acute phase due to elevated SAA concentration[16]. In addition, as mentioned before, SAA can bind to cell scavenger receptors to promote lipid internalization and contributes to tissue repair [36–38]. However, it may cause lipid accumulation in arterial macrophages, compounding atherosclerosis [58]. Based on such functions in the inflammatory process, SAA may be critical in the pathophysiology of numerous immune diseases, like inflammatory rheumatic diseases, cancers, and IBD [20, 59].

## SAA in the pathogenesis of IBD

Although the specific mechanisms remain inexplicit, it is generally accepted that IBD is associated with gene polymorphisms, the gut barrier, and immunity [7]. SAA can exert an important influence on the intestinal mechanical barrier, immune barrier, and gut microbiota by inducing cell differentiation, promoting gene expression and pathway activation, and enhancing intestinal antibacterial effects (Table 1).

**Table 1:** Functions of different SAAs in IBD.

SAA species	Related mechanism	Model	Functions	Reference
Mouse SAA3	Gut mechanical/ immunologic barrier	SAA3−/− mice with DSS-induced colitis	SAA3−/− contributes to shortening of the colon, loss of crypts, and decreasing regeneration of epithelium. SAA3-treated neutrophils increasingly secrete IL-22, promote secretion of the antimicrobial peptides and improve colonic epithelial integrity.	[63]
Human SAA1	Gut immunologic barrier	Human HT-29 and Caco-2 cells	SAA1 induces expression of IL-8 and COX-2. The promoting effect on IL-8 is depended on NF-κB, MAPK, and AKT pathways.	[45]
Mouse SAA3	Gut immunologic barrier	C57BL/6 and IL-1Rα−/− mice	SAA3 promotes IL-1β secretion of macrophages with the assistance of NLRP3 inflammasome and TLR2	[46]
Mouse SAA1/2	Gut immunologic barrier	SAA1/2−/− mice with AOM/DSS-induced colitis-associated cancer	Insufficient SAA1/2 alleviates colitis and tumorigenesis, and causes IL-4, IL-10, TNF-α decreased and lesser macrophages in distal colon, as well as SAA3 down-regulated	[21]
Mouse SAA1.1/2.1	Gut immunologic barrier	J774 cells	SAA1/2 promotes IL-1β expression via NLRP3 inflammasome, and this process can be inhibited by HDL	[47]
Mouse SAA1	Gut immunologic barrier	Mouse naive CD4 + T cells	SAA1 enhances the differentiation into TH17 cells depending on TGF-β	[75]
Mouse SAA1; Human SAA1/2	Gut immunologic barrier	Human cord blood naive CD4 + T cells; Mouse naive CD4 + T cells	SAA1/2 promotes pathogenic TH17 cells differentiation directly with the help of STAT3-activating cytokines	[14]
Mouse SAA1/2/3	Gut immunologic barrier	SAA1/2/3−/− mice	Inadequate SAA1/2/3 leads to attenuated colitis and lesser TH17 cells	[14]
Mouse SAA1/2	Gut immunologic barrier	SAA1/2−/− mice with DSS-induced colitis	SAA1/2−/− mice showed significantly decreased hematocrit, shortened colon and increased transcription of TNF-α and osteopontin compared with controls	[78]
Human SAA1	Gut microbiota	Gram-negative bacteria	SAA1 binds to OmpA on Gram-negative bacteria with high affinity and speed, and HDL cannot inhibit such binding	[80]
Mouse SAA1	Gut immunologic barrier/microbiota	Human macrophages and neutrophils	SAA1 promotes the phagocytosis of neutrophils and macrophages on Gram-negative bacteria. SAA1 promotes macrophages to produce more IL-10 and TNF-α	[70]
Mouse SAA2.2	Gut microbiota	Murine CMT93 and human HT-29 cells	SAA2 treatment inhibits the growth of co-cultured *E. coli*	[78]

*DSS* dextran-sodium-sulfate, *HDL* high-density lipoprotein, *TH* T helper, *TLR* toll-like receptor, *SAA* serum amyloid A.

### SAA and gut mechanical barrier

**Fig. 2:**
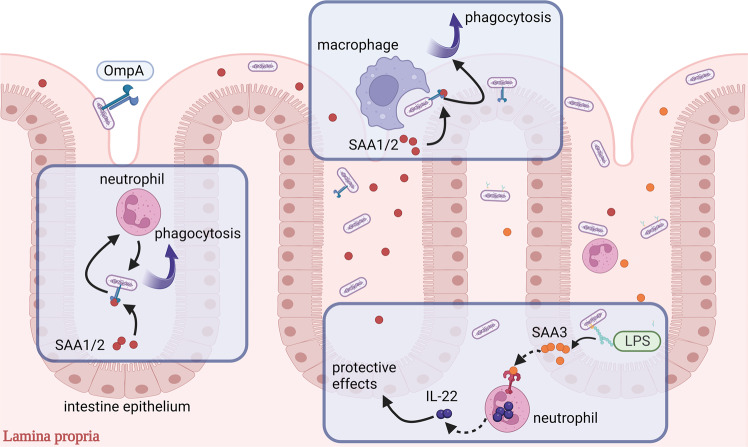
SAA participates in gut barrier. The lipopolysaccharides (LPS) expressed by Gram-negative bacteria induce the SAA3 secretion of epithelial cells. SAA3 binds to toll-like receptor 2 on the surface of neutrophils, promoting their IL-22 secretion, which performs a protective effect on the gut epithelium. SAA1/2 binds to OmpA and its homologs on the surface of Gram-negative bacteria, promotes the phagocytosis of neutrophils and macrophages on these bacteria.

The gut mechanical barrier consists of a continuous gut epithelium and junctions occluding the intercellular space, known as tight junctions. The mechanical barrier regulates the permeability of the bowel, which contributes to IBD [60]. In IBD, the gut mechanical barrier shows epithelial leakage, excessive apoptotic cell shedding, and increased permeability [60–62]. SAA has a protective effect on the intestinal mechanical barrier (Fig. 2). In dextran-sodium-sulfate (DSS)-induced colitis, the SAA level is extremely high, particularly in studies on mouse SAA3 [43, 63]. Furthermore, SAA3 deficiency of SAA3 knockout mice causes more serious DSS-induced injury, including shortening of the colon, loss of crypts, and decreasing regeneration of epithelium. In contrast, SAA3 treatment restored colonic length, alleviated weight loss and protected the crypts from damage [63]. In addition, colonic levels of Reg3β and Reg3γ antimicrobial peptides were increased after SAA3-treated neutrophils transfer, which can bind to bacterial peptidoglycans and defend against Gram-positive bacterial infection, thereby safeguarding the colon mechanical barrier [63]. The mechanism behind this is that SAA3 upregulates IL-22 expression in colon mucosal neutrophils through a TLR2-dependent signal pathway, which can restrain chronic inflammation [63–67]. Interestingly, rectal expression of SAA3 is significantly increased by IL-22 [68].

### SAA and gut immunologic barrier

The gut immunological barrier is made up of immunoglobulins secreted by plasma cells, lymphatic nodules in the mucosa, and immune cells dispersed in the lamina propria [69]. During IBD, the behaviors of cells that mediate both innate and adaptive immunity are altered. These cells include neutrophils; macrophages; innate lymphoid cells, and TH1, TH2, TH17 immune cells are all affected [6, 7].

**Fig. 3:**
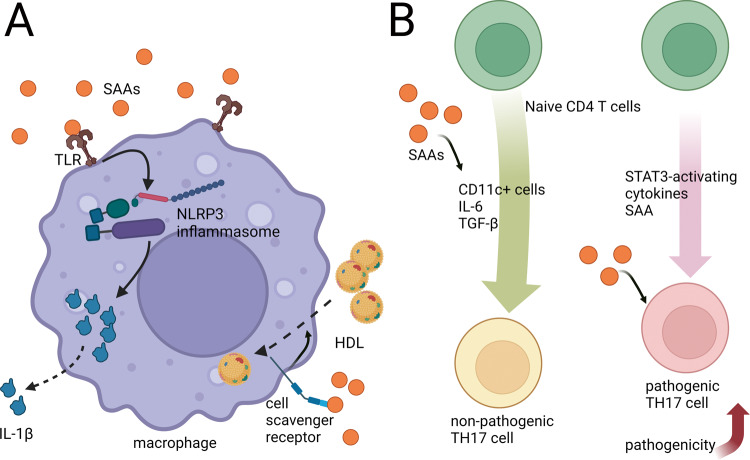
SAA acts on immune cells. **A** For macrophages, SAA can bind to toll-like receptors to activate NLRP3 inflammasome, then promote the secretion of IL-1β. SAA also binds to cell scavenger receptors to accelerate the uptake of lipids, facilitating tissue repair. **B** SAA enhances the differentiation of naive CD4 + T cells into non-pathogenic TH17 cells mediated by TGF-β, IL-6, and CD11c+ cells. SAA could directly act on naive CD4 + T cells, inducing differentiation into pathogenic TH17 cells with the help of STAT3-activating cytokines.

In innate immunity, SAA mainly influenced macrophages (Fig. 3A). Lesser distal colonic infiltration of macrophages was found in SAA1/2 double-knockout mice with colitis than in wild-type ones [21]. Functionally, SAA1 can bind to cell scavenger receptors expressed on macrophages to promote lipid isolation, removal, and circulation during inflammation, assisting tissue repair [36–38]. SAA can also intensify phagocytosis, contributing to the killing of Gram-negative bacteria by macrophages and neutrophils, inhibiting bacterial pro-inflammatory processes [70, 71]. Furthermore, SAA treatment activates caspase-1 and markedly aggrandizes pro-inflammatory IL-1β release from macrophages [47, 48]. Similarly, in SAA-treated keratinocytes, the transcriptional levels of caspase-1 and NLRP3 were both obviously increased. Thus, SAA is believed to induce IL-1β via the NLRP3 inflammasome, accelerating IBD progression [46–48, 72]. SAA can also work as a chemokine for neutrophils [73]. Importantly, in SAA3 knockout mice, lack of SAA3 resulted in lower serum and colon IL-22 level. Thereafter, colonic IL-1β, IL-6, and TNF-α were all significantly increased, suggesting that SAA3 deficiency can lead to more severe inflammation [63].

In adaptive immunity, SAA is closely associated with T lymphocytes, especially TH17 cells (Fig. 3B). SAA is a T-cell chemoattractant [74]. In network analysis, a direct positive correlation between SAA1 and TH17 cells was found, whereas SAA1 was negatively correlated with the abundance of TH22 cells [15]. In vitro experiment found SAA1 expression markedly enhances naive CD4 + T cells differentiating into TH17 cells, which is mediated by transforming growth factor β (TGF-β), IL-6, and CD11c+ cells, suggesting an association between SAA1 and TH17 cells [75]. Importantly, Lee et al. proposed another function of SAA on inducing the pathogenic transformation of pro-inflammatory TH17 cells independent of TGF-β signaling. Their in vitro experiment showed that SAA collaboration with IL-6 can perform a prominent inductive effect on the differentiation of pathogenic pro-inflammatory TH17 cells, even with the existence of a TGF-β antibody. Deeper research on CD4 + T cells has found that SAA augments IL-6-mediated IL-23 receptor induction, which translates into stronger STAT3 activation and promotes the differentiation of T cells [14]. In *Helicobacter hepaticus*-colonized SAA1/2/3 tri-deficient mice, the antibacterial responses 2 weeks after transferring CD4 + T cells were similar to controls, manifesting that SAAs do not influence the differentiation of T cells during homeostasis. Sustained IL-10 receptor A blockade causes augmentation of pro-inflammatory TH17 cells and deteriorating colitis [14, 76, 77]. Then, 5 days after transfer, decreased numbers of TH17 cells in the mesenteric lymph nodes and TH1 and TH17 cells in the colonic lamina propria were found in SAA1/2/3 tri-deficient mice, and their colitis histologic features were also attenuated compared to controls. Together, these results demonstrate the inductive effect of SAA on pro-inflammatory TH17 lymphocytes and its contribution to colitis [14]. Thus, in cooperation with STAT3-activating cytokines, SAA directly induces naive CD4 + T lymphocytes to differentiate into pathogenic TH17 lymphocytes, rather than the non-pathogenic TH17 lymphocytes mediated by TGF-β [14, 75]. Researches on transgenic mice with experimental autoimmune encephalomyelitis indicated that SAAs can promote TH17-mediated inflammatory diseases by enhancing the pathogenicity of activated TH17 cells [14]. Although an experiment using SAA1/2 double-knockout mice with DSS-induced colitis had produced similar results [21], another animal study with the same model had come to an opposite conclusion as SAA helps killing Gram-negative bacteria [70, 78]. This leads us to believe that the pro-inflammatory and protective effects of SAA are in a dynamic competitive relationship. Examination of biopsy specimens from UC patients showed that epithelial cells and lamina propria cells in areas neighboring to inflammation significantly expressed SAA1/2, whereas cells in areas adjacent to non-inflammation did not [14]. The same phenomenon was observed in CD patients [78]. Accordingly, SAA modulates the generation and activity of TH17 lymphocytes, enabling itself to be a potential therapeutic target for IBD treatment [14].

### SAA and gut microbiota

The gut microbiota is demonstrated to have a bidirectional association with IBD progression. Gut microbiota shows decreased microbial diversity, decreased abundance of Firmicutes, and increased abundance of Gram-negative Proteobacteria in IBD [79]. Gram-negative bacteria are targets for SAA to exert antibacterial effects, which may work against IBD progression [70, 71, 80, 81].

Antibacterial effects of SAA occur mainly by binding outer membrane protein A (OmpA) and its homologs on the outer membrane of Gram-negative bacteria (Fig. 2). SAA1 has been observed to bind to Gram-negative bacteria, such as *Escherichia coli* and *Salmonella typhimurium*. These bindings have a high affinity, are fast, and cannot be inhibited by HDL [80]. Importantly, OmpA was found in the outer membrane of *E. coli* and showed an antibacterial effect through SAA. Supporting evidence showed that *E. coli* without OmpA expression was not affected by SAA. Ligands in other bacteria are likely OmpA homologs [80]. On this basis, another experiment showed that after SAA conditioning, the phagocytosis of neutrophils on *E. coli* was strengthened significantly. Meanwhile, the phagocytic effect of macrophages derived from peripheral blood monocytes on *E. coli* was also enhanced [70]. In conclusion, SAA expression contributes to the Gram-negative bacteria vanishing by phagocytes, obstructing the development of IBD, and this effect could occur at normal concentrations [70, 71, 81]. A controlled study further confirms conclusions mentioned above. In cultured murine rectal epithelial cell lines, SAA3 expression is strongly upregulated by bacterial lipopolysaccharides, whereas SAA1/2 expression is not [43, 63, 78]. SAA1/2 overexpression can restrain the viability of co-cultured *E. coli*, which may interpret the protective effects of SAA in DSS-induced colitis [70, 78]. Furthermore, in SAA1/2 double-knockout mice with DSS-induced colitis, significantly decreased hematocrit values and shortened colon were found compared to that of wild-type ones, convincing that SAA helps attenuate susceptibility of colitis [78].

It has been reported that SAA expression could be induced by bacterial-derived products. Specifically, bacterial lipopolysaccharides could promote SAA expression [43, 68]. In addition, the segmented filamentous bacteria adhering to mice intestinal epithelial cells could cause an accumulation of TH17 cells in the lamina propria, whose SAA1-3 transcriptions are significantly upregulated [82]. Further studies of this system revealed that following the adhesion of segmented filamentous bacteria, IL-22 was released by type 3 innate lymphoid cells in the ileum, and SAA1/2 was produced through a STAT-independent mechanism [75, 83]. The upregulation of SAA could be a response to abnormal bacterial activities, inhibiting pro-inflammatory processes. However, this may contribute to the progression of IBD, considering the regulation of gut immunological barrier of SAA [14, 83]. Further research in this area is required.

## SAA in patient management of IBD

Endoscopy is the gold standard for assessing disease activity in IBD and is one of the most important tools for disease diagnosis and patient stratification [84, 85]. However, owing to its invasiveness, time consumption, and high cost, regular endoscopy procedures are rarely performed on patients. Therefore, biomarkers, as noninvasive, low-cost, and accurate indicators, have received considerable attention in patient management in IBD [86]. SAA is involved in critical processes of IBD pathogenesis and may become a promising biomarker in clinical practice. In this section, we summarize the research focusing on SAA and the diagnosis, disease activity assessment, prognosis prediction, and treatment of IBD (Table 2).

**Table 2:** SAA in patient managements of inflammatory bowel disease.

Patients	Outcomes	Samples	Performance	Reference
Diagnosing disease				
23 healthy controls; 8 controls with non-IBD disease; 67 UC	–	Biopsies come from the terminal ileum, ascending colon, descending colon, and sigmoid colon	Fold change: 8.18	[87]
21 healthy controls; 106 UC	–	Biopsies come from mucosa	AUC = 0.8097	[88]
27 healthy controls; 105 IBD	–	Biopsies come from colon, inflamed ileum and inflamed rectum	Fold change >1.5	[89]
20 healthy controls; 118 IBD	–	Serum samples	Higher SAA concentration in IBD patients (*P* = 0.005)	[90]
Assessing disease activity				
55 CD	Clinical remission: CDAI <150	Serum SAA concentration	Tenfolds lower in remission	[91]
43 CD; 52 UC	Clinical activity: HBI >4	SAA concentration	Fourfolds higher in activity	[92]
55 CD	Endoscopic remission: SES-CD ≤3	Serum SAA concentration	AUC = 0.77	[91]
94 CD	Endoscopic remission: no intestinal mucosal inflammatory findings	Serum SAA concentration	AUC = 0.77	[93]
108 UC	Mucosa healing: MES 0 or 1; Complete mucosal healing: MES 0	Serum SAA concentration	Complete mucosal healing: AUC = 0.794	[94]
36 CD; 35 UC	Endoscopic remission: CD: SES-CD 0-3; UC: MES = 0	Serum SAA, IL-6, IL-8, and Eotaxin-1	AUC = 0.84	[90]
94 CD	Histologic remission: no lamina propria neutrophils, lymphoid aggregation and basal plasmacytosis	Serum SAA concentration	AUC = 0.81	[93]
Predicting prognosis				
41 CD	Relapse: CDAI ≥150	Serum SAA concentration ≥5.9 μg/dl	SAA concentration ≥5.9 μg/dl is associated with a higher rate of relapse	[91]
32 UC	Endoscopic remission: MES 0 or 1	Serum SAA concentration	Lower SAA concentration at week 14 is associated with endoscopic remission	[96]

*AUC* area under the ROC curve, *CD* Crohn’s disease, *CDAI* CD activity index, *IBD* inflammatory bowel disease, *MES* Mayo endoscopic score, *SAA* serum amyloid A, *SES-CD* simple endoscopic score for CD, *UC* ulcerative colitis.

### Diagnosing disease

Due to high expression of SAA during the acute phase of illness, intestinal SAA expression increases significantly during inflammation [12, 13], which can be used as an indicator for the diagnosis of IBD. A series of researches have uncovered that the SAA gene level, mainly SAA1/2, is upregulated in patients with UC. A genome-wide expression study of intestinal biopsies revealed that SAA1 was the most elevated gene with a fold change of 8.18 when compared to control participants [87]. Moreover, a weighted gene coexpression network analysis of 127 biopsy samples from UC patients and healthy controls showed that SAA1/2 are potential genetic biomarkers for the diagnosis of UC, with an area under the ROC curve (AUC) of 0.8097 (95% confidence interval [CI]:0.6975-0.9220) [88]. In both CD and UC, the high expression of SAA2 in inflamed locations has been demonstrated by Jason et al., with a fold change of more than 1.5 when compared to participants without IBD [89]. In addition to SAA genes, serum SAA levels in IBD groups exceeded that in healthy controls. However, its diagnostic ability has not yet been studied [90].

### Assessing disease activity

Considering the critical function of SAA during inflammation, it is not surprising to find that its expression is in parallel with IBD disease activity, including clinical, endoscopic, and histologic activity. Serum SAA was demonstrated to be associated with the Crohn’s disease activity index with a Spearman correlation coefficient of 0.42. The serum SAA concentration was 10-fold lower in CD patients with clinical remission than in those with clinical activity [91]. Another study that included 43 CD and 52 UC patients also found higher concentrations of SAA in active patients [92]. Regarding endoscopic activity, several researchers revealed the promising predictive ability of serum SAA in both CD and UC [90, 91, 93, 94]. Ishihara et al. recruited 55 CD patients and revealed a positive correlation between serum SAA level and endoscopic activity, with an AUC (95% CI) of 0.77 (0.64–0.90) for identifying endoscopic remission [91]. Another research study with 94 CD patients demonstrated that SAA had a higher AUC (0.77 vs 0.75), sensitivity (0.676 vs. 0.558), and specificity (0.960 vs. 0.692) for predicting endoscopic activity than C-reactive protein (CRP), an extremely vital biomarker for assessing disease activity in IBD [93, 95]. Moreover, SAA can distinguish 70% of endoscopic activity patients with relatively low CRP concentrations [93]. A study including 199 endoscopy procedures in patients with UC showed that serum SAA correlates with endoscopic activity mightier than CRP (correlation coefficient 0.641 vs. 0.352). The AUC of SAA for detecting endoscopic activity was 0.794, which was higher than that of CRP (0.646) [94]. Furthermore, the ability of SAA to identify endoscopic activity in IBD can be improved by combining it with other inflammatory biomarkers [90]. Bourgonje et al. built a model based on SAA, eotaxin-1, IL-8, and IL-6 to identify endoscopic activity in patients with IBD. This model had an AUC, specificity, and sensitivity of 0.84, 0.684, and 0.907, respectively, to predict endoscopic activity [90]. Only one study focused on histologic activity in patients with CD [93]. In this study, SAA could predict histological remission with an AUC of 0.81, which was superior to that of CRP. Additionally, SAA combined with other biomarkers, such as IL-6, IL-8, and TNFα, would have a higher AUC to identify histological remission [93].

### Predicting prognosis

The SAA level has been proposed as a possible prognostic factor for IBD outcomes, such as relapse and therapeutic outcomes [91, 96]. One study recruited 41 CD patients with clinical remission and found that higher SAA concentration, defined as more than 5.9 μg/dl, indicated more rapid incidence of disease relapses [91]. In addition, SAA was demonstrated to be associated with endoscopic outcomes after receiving vedolizumab in a prospective study of 32 patients with UC [96]. In this study, the SAA concentration declined dramatically from baseline (45.5 μg/ml) to week 14 (3.6 μg/ml) during vedolizumab therapy. The week-14 SAA level was lower in endoscopic remitting patients, while a prominent relationship between SAA and clinical remission was not discovered. Moreover, the SAA levels at weeks 2 and 6 were not associated with clinical or endoscopic remission. At week 26, the SAA concentration seemed to decrease, especially in patients who achieved either clinical or endoscopic response. However, there was no significant difference, which may have been limited due to the sample size [96].

### Potential therapeutic target

Although effective anti-inflammatory biological drugs, such as anti-TNF, anti-integrin, and anti-p40 subunit antibodies, are already used in IBD, these drugs are expensive and sometimes lack a response. However, inexpensive and effective oral drugs remain elusive [97, 98]. Recently, 5-MER peptide (5-MP) methionine-threonine-alanine-aspartic acid-valine, a drug that binds specifically to SAA, has been discovered [99]. 5-MP disrupts both hexamer assembly and amyloid aggregation of SAA, attenuating its pro-inflammatory activity. Such binding significantly suppresses the secretion of pro-inflammatory cytokines by SAA-activated cells. In contrast, protective genes against chronic inflammation and neuronal degeneration were upregulated after treatment with 5-MP. Importantly, the use of 5-MP would not lead to anti-drug antibodies and disruption of the normal immune response [99]. Animal experiments showed 5-MP inhibits chronic inflammation in SAA-dependent diseases, such as collagen-induced arthritis, autoimmune encephalomyelitis, and IBD [99]. In tri-nitro-benzene-sulfonic acid-induced IBD mice, 5-MP was no less effective than anti-TNF therapy. After 5-MP treatment, the colon transcriptions of IL-1β and IL-6 significantly decreased, while interferon-γ was upregulated [99]. Although the efficacy and safety of 5-MP in IBD patients required further evaluation by more randomized controlled trials, the emergence of 5-MP provides new insight into the therapeutic targets of IBD. We believe that with further research, it is possible to find more precise targets, such as the receptors of SAA, or different subtypes of SAA, and possibly achieve better efficacy and safety to optimize IBD treatment.

## Conclusion

In this review, we focused on SAA, summarized its structure and functions, and their role in the pathogenesis and treatment of IBD. Four helices and an amorphous C-terminal tail exist in the SAA monomer. Different sites can bind to different ligands, leading to multiple functions and important roles in IBD. SAA plays a variety of roles in IBD, including protecting the intestinal epithelial barrier, inducing T-cell differentiation, antibacterial effects, etc., which make it a potential biomarker for IBD. In recent years, the close relationship between SAA and IBD has been explored by a growing number of studies. SAA, comparable to CRP, could be used to help manage IBD, including diagnosis, assessment of activity, and prognosis. Moreover, SAA could be helpful in optimizing treatment decisions and finding new treatment options, thus further improving the management of IBD.

It is important to note that SAA exhibits a duality of inflammation during both the acute and chronic phases. Considering IBD as a chronic inflammatory disease, it is not surprising to observe the duality of SAA in it. This duality may be related to the type of SAA. From the studies we list in Table 1, we found SAA3 is more protective by acting on neutrophils to maintain the integrity of gut epithelium. As for SAA1/2, although they also show anti-inflammatory effects through binding to HDL to accelerate tissue repair and promote phagocytosis on Gram-negative bacteria, their pro-inflammatory effects on immune cells are even more pronounced. The combined effect of SAA1/2/3 remains pro-inflammatory, which may account for the treatment potential of anti-SAA therapy in IBD, though further research is needed. However, whether there is any crosstalk between SAA3 and SAA1/2 is unclear. It is still a worthwhile line of future inquiry. Furthermore, SAA concentration may also play a role in the duality, but there is not enough information to support this claim. Hopefully, with a deeper exploration of the role of SAA in IBD, more precise targets will be found to improve the IBD treatment.

## Data Availability

All data included in this review are available upon request by contact with the corresponding authors.
